# Implementing an online-delivered exercise program for childhood cancer survivors: A hybrid effectiveness-implementation protocol for the MERRIER study

**DOI:** 10.1016/j.jsampl.2025.100095

**Published:** 2025-03-12

**Authors:** David Mizrahi, Alexandra Martiniuk, Laurence Hibbert, Dinisha Govender, Tora Sibbald, Richard Mitchell, Natalia Millard, Lauren Ha, Damian Ragusa, Kylie Brown, Ben Smith

**Affiliations:** a The Daffodil Centre, The University of Sydney, A Joint Venture with Cancer Council NSW, Sydney, Australia; b Sydney School of Public Health, Faculty of Medicine and Health, The University of Sydney, Australia; c Cancer Voices, Sydney, Australia; d Cancer Centre for Children, Children's Hospital at Westmead, Sydney, Australia; e Kids Cancer Centre, Sydney Children's Hospital, Sydney, Australia; f Discipline of Paediatrics & Child Health, School of Clinical Medicine, UNSW Medicine & Health, University of New South Wales, Sydney, Australia; g Services & Programs, Camp Quality Australia, Sydney, Australia

**Keywords:** Childhood cancer, Paediatric oncology, Exercise, Physical activity, Fitness, Digital health

## Abstract

**Background:**

Physical activity levels are low in childhood cancer survivors. Structured physical activity programs are not routinely provided, despite being safe and beneficial for improving physical and psychological health. Innovative health promotion programs delivered online may allow families to receive equitable health support, which may foster survivors to improve their health.

**Aims:**

To determine implementation factors of an online exercise program recruited through a community organization, and effectiveness on physical activity levels and self-efficacy for childhood cancer survivors.

**Methods and analysis:**

The MERRIER study is a type-1 hybrid effectiveness-implementation. Sixty children (5–18 years old) who have completed treatment for any cancer type will be enrolled between March 2025 and June 2026. Participants will be randomised (stratified by age, cancer type and sex) 1:1 to 3-months multimodal exercise or control group. The intervention group will receive five online consultations with an Accredited Exercise Physiologist to provide behaviour counselling, and prescribe an individualised aerobic, resistance and balance exercise program at low-moderate intensity. The RE-AIM framework will assess reach (e.g. recruitment rate), effectiveness (e.g. physical activity levels), adoption (e.g. qualitative interviews), implementation (e.g. exercise adherence), and maintenance (e.g. self-efficacy at follow-up). Physical function and patient-reported outcomes will be assessed at baseline (T0), post-intervention (T1; week 12) and follow-up (T2, week 24). An Axivity AX3 accelerometer will measure physical activity over five-days at T0/T1.

**Implications:**

If effective, we aim to collaborate with community organisations, who are well placed to implement similar programs to childhood cancer survivors.

**Ethics:**

The study was approved by The University of Sydney Health Research Ethics Committee (2024/HE000391).

**Trial registration:**

ACTRN12624000604505p.

**Key points**

- This is the first study to investigate the implementation and delivery of an online exercise program to childhood cancer survivors in conjunction with a community organisation.
- Children aged 5–18 will be provided with individualised exercise programs based on their needs, preferences and abilities.
- If successful, this online program could be an equitable option to deliver programs to children, which is particularly useful to families who live far from specialised services.

## 1 Introduction

The survival rate of childhood cancer is improving due to improved treatment protocols and supportive care [1,2]. However, the cost of cure is high, with most survivors experiencing lifelong chronic conditions [3]. Treatment protocols for paediatric oncology often include therapies such as anthracycline, methotrexate, vincristine, steroids and thoracic radiation [1]. These therapies can increase risks of conditions such as cardiovascular disease, diabetes, obesity, osteoporosis, mental health disorders and secondary cancers [4]. Yet, though childhood cancer survivors have increased risk for several chronic conditions they also are less likely to engage in physical activity and have lower fitness than their same aged peers [2,5] further increasing their risk and burden caused by these conditions.

Participating in regular physical activity and exercise can improve fitness, muscle strength, metabolic health, bone density, fatigue, mental health and quality of life for children impacted by cancer [2,[6], [7], [8]]. Participating in regular exercise has also demonstrated to reduce the risk of cardiovascular, metabolic, and psychological adverse and late-effects of childhood cancer treatment [9,10]. Recent international paediatric oncology exercise guidelines [11] called for exercise and physical activity to be promoted across all key settings of childhood cancer survivors’ lives, including hospitals, homes, schools and communities. Despite these benefits and guidelines, most childhood cancer survivors participate in less physical activity than their peers [12] and may benefit from physical activity support. It is particularly important to establish healthy behaviours in childhood [13], it is crucial to support, motivate and educate childhood cancer survivors to become active to maintain their health and reduce the risk of developing chronic conditions [3].

Identifying and overcoming barriers to implementing exercise and physical activity programs into care is an important next step to improve short and long-term physical and psychological health of childhood cancer survivors [14]. Several barriers prevent childhood cancer survivors from receiving physical activity programs. Families may experience practical barriers such as not living locally to specialised services, lack awareness of the benefits, or have financial constraints [15,16]. Healthcare providers may be a facilitator or barrier in motivating patients to become physically active [15]. Organizational barriers including time and staff resources, and lack of established referral pathways have been identified as key barriers to implementing exercise programs in oncology [17,18]. In Australia, while some oncology multi-disciplinary teams include exercise professionals such as Accredited Exercise Physiologists (AEP) and physiotherapists, a lack of widespread implementation means that not all patients can be supported [19].

Physiotherapists (also termed physical therapists) and AEPs (also termed certified exercise physiologists, kinesiologists) are well-suited exercise professionals to provide assessments, advice, programs and self-management on exercise. These allied health professionals may play an important role in engaging childhood cancer survivors in physical activity and coaching them to obtain the tools to self-motivate themselves. Exercise programs can be individualised for childhood cancer survivors based on physical ability, needs, exercise history and preferences to ensure adequate adherence. Given the heterogeneity and health challenges the childhood cancer population experience, the MERRIER study has been developed to support these children engage in tailored physical activity programs and build their self-efficacy.

There is growing recognition among families of childhood cancer patients and healthcare providers to access programs to support physical recovery after treatment, to address their unique physical and psychosocial challenges. In an Australian study, among 64 parents of childhood cancer survivors who received an exercise physiology consultation, 97 ​% felt that future children with cancer would benefit from an assessment by an AEP [20]. In Australia, the publicly funded Medicare system allows eligible people with a chronic condition to receive up to five subsidized consultations per year with AEPs or physiotherapists. Despite potential benefits, there are challenges to delivering assessment and supports from AEPs and physiotherapists due to scheduling and geographic constraints [21]. This has created the need to connect with families in innovative ways, such as using online and remote digital health services and connecting with local community organisations. Recent studies have demonstrated the validity of performing remote physical assessments online in teenagers and young adults with and without mobility disabilities [22], which is useful in countries like Australia where patient populations often live far from specialised services [21]. Additionally, delivering exercise support programs using online platforms has been demonstrated to be feasible in childhood cancer survivors, and able to achieve acceptable program adherence [7]. While there are remotely delivered exercise trials in progress among young adult survivors of childhood cancer (aged 18–39 years) [23], there are limited studies conducted in children and adolescents aged 5–18 years. Additionally, there are limited paediatric exercise oncology studies simultaneously evaluating the effectiveness of exercise while identifying barriers and facilitators to its implementation [24].

### 1.1 Aims

This trial is a type 1 hybrid effectiveness-implementation trial designed to:

1) Determine the effectiveness of the MERRIER intervention, an exercise program delivered online to childhood cancer survivors in conjunction with a community organisation, in improving moderate-vigorous physical activity levels and physical activity self-efficacy.
2) Examine the factors that may increase the program reach, effectiveness, adoption, implementation and maintenance

## 2 Methods and analysis

### 2.1 Design and setting

A mixed methods type 1 hybrid effectiveness-implementation randomised trial design will be used to evaluate the preliminary effectiveness and implementation of the “Maximizing Exercise, Resilience, and Implementation to Empower Recovery – An Online Intervention for Increasing Physical Activity and Self-Efficacy in Childhood Cancer Survivors” (MERRIER) study (ANZCTR Trial Registration #ACTRN12624000604505p) [25]. The MERRIER study will be delivered by researchers with recruitment facilitated by a childhood cancer community organisation (e.g. Camp Quality).

### 2.2 Participants and screening

Recruitment is planned between March 2025 and June 2026. Eligibility criteria for participants include those: aged 5–18 years, diagnosed with any cancer type, completed intensive treatment (e.g. intravenous chemotherapy, stem cell transplant, radiation therapy, however maintenance therapy is permitted). Participants and a parent/guardian must have sufficient English proficiency to understand the consent process and exercise intervention. Translation services are available for participants who speak supported languages, as specified by service availability. Exclusion criteria include having a medical condition that would prohibit exercise participation at discretion of the research team and if needed in consultation with treating doctor (e.g. but not limited to cardiomyopathy, uncontrolled cardiac arrhythmias), and pregnant females.

Parents of potential participants will be informed of the opportunity to participate by the community organisation staff. Parents/guardians of eligible participants will be provided with the study information sheet and consent form to consider permitting their child to participate. Families from outside community organisations are permitted to enrol. Parents will then discuss the study in more detail with the lead investigator (DM) prior to providing informed consent. Consent forms and study data will be then collected and managed by the research team using Research Electronic Data Capture (REDCap) [26], who will then become the point of contact for the study. After providing informed consent, information will be gathered about cancer-related medical history, treatment-related side effects, other chronic conditions or injuries, and physical activity readiness and risk-management via the Pre-Exercise Screening System for Young People (PSS-YP) [27]. The intake form and PSS-YP will be reviewed by the lead investigator (DM), who is an Accredited Exercise Physiologist accredited with Exercise and Sports Science Australia, to conduct a safety screen for exercise participation.

### 2.3 Randomization

**Fig. 1:**
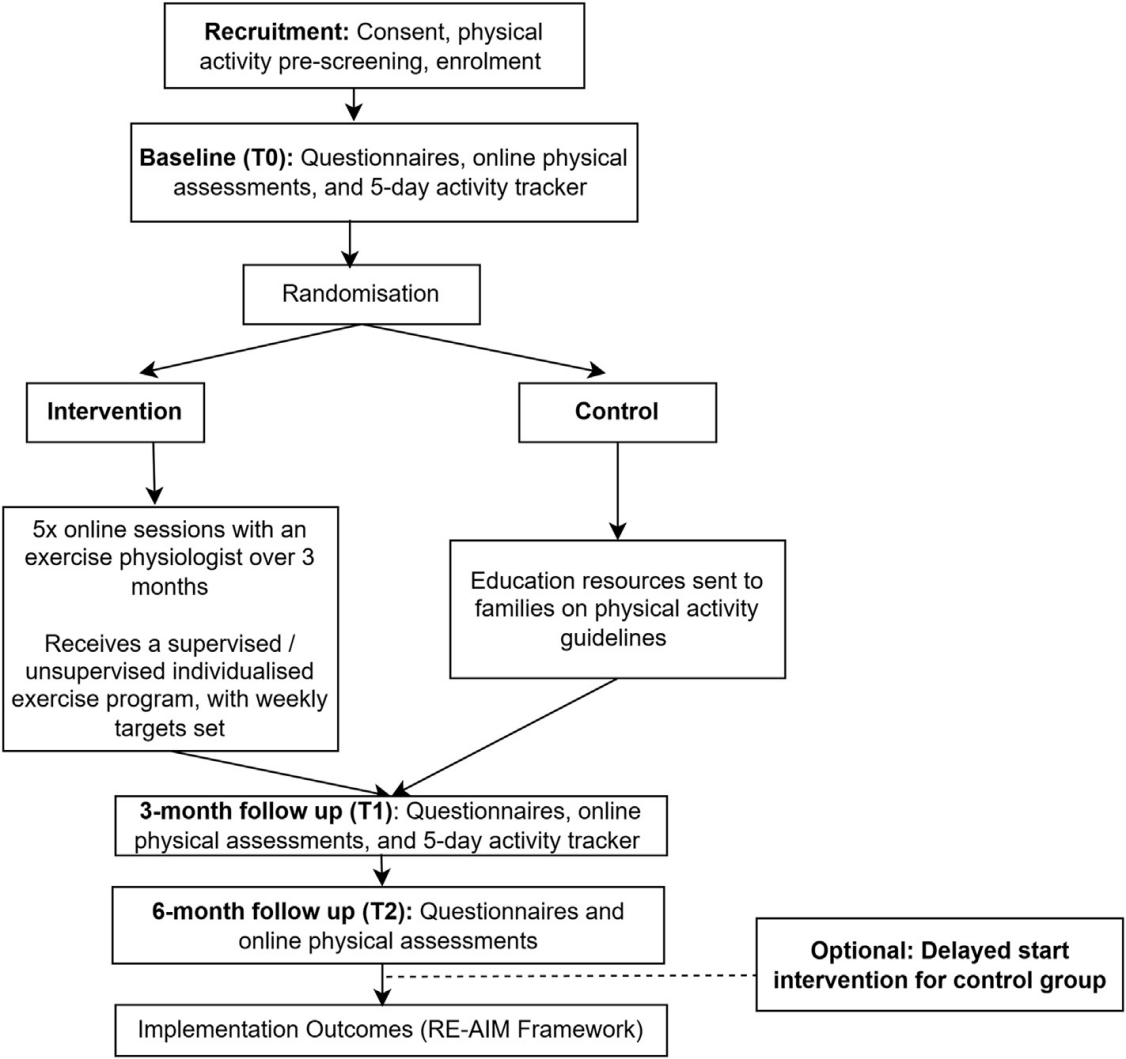
Flow chart of study procedures.

Following the completion of baseline (T0) assessments including physical assessments, questionnaires and 5-day objective physical activity wrist-worn accelerometry, participants will be assigned to either the exercise intervention or control group (Fig. 1). Randomization will occur in a 1:1 ratio using the online computer-generated randomisation software, Sealed Envelope, which will be completed by an independent researcher outside the core study team. Randomisation will be performed in blocks to ensure balance across important variables, including age (categorized into three groups: 5–9, 10–13, and 14–18), sex (male, female), and disease type (liquid, solid, brain tumours).

### 2.4 Outcome measures

Outcome measures are presented in Table 1, and will be completed at three time points: (1) baseline (T0; week 0); (2) post-intervention (T1; week 12); and (3) follow-up (T2; week 24). Physical assessments are conducted online using Microsoft Teams by an AEP in the research team. Patient-reported outcomes (PROs) questionnaires will be emailed to each participant's parent/guardian at all time points via REDCap. Accelerometers will be mailed to participants to be worn for 5-days at the baseline (T0) and post-intervention (T1) timepoints.

**Table 1:** Summary of study timepoints and procedures.

	Baseline (Week 0)	Post-intervention (Week 12)	Follow-Up (Week 24)
**Accelerometer:** Physical activity level (wrist-worn accelerometer for 5-days)	x	x	
**Physical assessments:**			
Sit-to-stand	x	x	x
Push up test			
Timed up-and-go			
Balance			
2-min step test			
Sit and reach			
**Questionnaires:**			
Physical activity self-efficacy (PASES survey)	x	x	x
Physical activity level (Godin's LSI)			
Quality of life (EQ5D5L)			
Self-reported fitness (IFIS)			
Fatigue (PedsQL-FMS)			
Implementation outcomes (e.g. Acceptability, RE-AIM framework)			x

### 2.5 Physical fitness outcomes

Physical fitness assessments will be conducted online using Microsoft Teams at each time point, and will take approximately 20–30 ​min, with results recorded in REDCap. All assessors will be trained in the assessment protocol, with the lead researcher having paediatric exercise oncology experience. Assessors will send videos of each assessment prior to the session, and then explain and demonstrate each assessment prior to the participants’ attempt. The researcher will record the results and supervise participants, in addition to having a parent or guardian supervising them in person, unless mutually agreed that parent supervision is not required (for adolescent participants). The physical fitness assessments (Table 1) include the following validated assessments.

#### 2.5.1 Sit to stand

The 30 ​s sit-to-stand (STS) test is a measure of lower body strength, measures the number of sit-to-stand repetitions completed over a 30 ​s period [28], which the researcher will count. The higher number of sit-to-stands performed is indicative of greater lower body strength.

#### 2.5.2 Push up

A 30 ​s push up test will be used to measure upper body strength, with the total number performed measured [29]. Participants will perform the same type of push up at all assessments, which will be a full (toes on floor) or half (knees on the floor) push up.

#### 2.5.3 Timed up and go

Timed up and go (TUG) test assesses physical function, by recording the time a patient needs to get up from a chair, walk 3 ​m, turn around, walk back, and sit down again [30]. Participants will be provided with a pre-cut 3-m rope. This is measured in seconds with a handheld stopwatch, with participants performing the TUG two times, with the mean of these measurements then calculated.

#### 2.5.4 Balance

A single leg balance test will be conducted to assess static balance in two conditions: eyes open and eyes closed [31]. The participant will be placed standing near a wall, with arms crossed, and legs parallel to each other on a flat surface, with a parent/guardian standing beside them. Both sides will be assessed up to two times, with the highest score taken. Participants stop the test if their arms come off the crossed position, if their legs touch each other or begin to flail, or if they reach the maximum of 30 ​s.

#### 2.5.5 2-Min step test

This is a sub-maximal test of cardiovascular fitness [32]. Participants will stand with their right leg facing the camera, and instructed to march on the spot for 2 ​min. The participant is instructed to raise their knee height, so the thigh becomes parallel to the floor when marching. When the test starts, participants begin marching on the spot. The researcher will count the number of steps completed within the 2-min time frame on the leg facing the camera. Participants will report their rate of perceived exertion (RPE; 1–10) [33] after the assessment has been completed.

#### 2.5.6 Sit and reach

Sit and reach test is a measure of flexibility of the lower back and hamstring muscles, which has been extensively used and validated in childhood populations [34]. This test involves sitting on the floor with legs stretched out straight ahead. The participant holds that position for at least 2 ​s while the distance is recorded by their parent/guardian. Touching the toes equals the baseline (0 ​cm), with any performance past the toes indicating a positive score in cm, and not reaching the toes indicates a negative score in cm. Participants will be provided with a ruler to measure the distance from the toes.

### 2.6 Patient-reported outcomes

Participants will complete questionnaires online in REDCap at baseline (T0), post-intervention (T1), and follow-up (T2). All included questionnaires are validated for completion by children of 5 years of age (except the EQ-5D-Y-5L which is from 8 years). Parents will be encouraged to assist their children in completing the questionnaires if required. Parents can also complete the questionnaires as proxy for their child particularly in the cases of cognitive dysfunction or unwillingness from participant.

#### 2.6.1 Self-efficacy

Physical activity self-efficacy will be measured via 8 items using the Physical Activity Self–Efficacy Scale (PASES), which has been validated for use among children and adolescents [35].

#### 2.6.2 Physical activity

Physical activity levels will be self-reported using Godin's Leisure Score Index, which is a four-item self-report questionnaire [36]. Three questions are stratified by intensity (low, moderate, and vigorous), and will be tallied for a global weekly score. We will ask participants for average minutes to calculate minutes per week for each intensity. This questionnaire has shown good reliability/validity in children and adolescents without cancer, with test-retest co-efficient ​= ​0.84 and previously used with childhood cancer survivors [37].

#### 2.6.3 Fitness

The International Fitness Scale (IFIS) developed by Ortega et al. (2011) assesses self-perceived fitness in adolescents, including cardiovascular, sprinting, muscle, flexibility and overall fitness, with answers using a 5-point Likert-scale [38].

#### 2.6.4 Quality of life

Quality of life will be measured using the EQ-5D-Y-5L, which is measure of health-related quality of life (HRQoL), and includes 5 items on a 5-point Likert scale relating to different domains of HRQoL and validated for use in children [39]. Additionally, we will use the EQ VAS, which a single measure asking participants to ‘rate your health between 0 and 100'.

#### 2.6.5 Fatigue

The PedsQL™ 3.0 Multidimensional Fatigue Scale™ (PedsQL-FMS) is a questionnaire consisting of 18 items describing symptoms of cancer-related fatigue. Each item is rated for how frequently it is a problem on a 5-point scale from 0 “almost never” to 4 “almost always”.

### 2.7 Objective physical activity levels

Average daily minutes of moderate-vigorous physical activity will be objectively measured using an Axivity AX3 accelerometer. The Axivity AX3 accelerometer is a research-grade waterproof wrist accelerometer that records continuous daily activity and captures acceleration along three axes (x, y, and z) with a sample frequency of 100 ​Hz. It shows good validity and accuracy at both wrist locations (ICC of 0.91 for comparison of left and wright wrists) [40], and validated with other research grade accelerometers with an ICC of 0.95 [41]. Participants will be instructed to attach the Axivity AX3 accelerometer on their non-dominant wrist for 5 consecutive days (including two weekend days and evenings). To be included for weekly physical activity calculations, at least four valid days are required. Valid days are defined as wearing the accelerometer for at least 10 ​h/day [40] with non-wear time being defined as not wearing the accelerometer for 60 consecutive minutes [41]. Accelerometer data will be collected at the start and end of the intervention. Participants will be provided with instruction on how to wear the accelerometer and will be provided with postage paid return envelopes for baseline (T0) and post-intervention (T1). Once returned to the researchers, participant data will be immediately downloaded onto a password protected computer.

### 2.8 Exercise intervention

The five exercise consultations are conducted online using Microsoft teams, and the exercise prescription is delivered remotely to participants using Physitrack (Physitrack Limited) exercise prescription software (available to participants as a website or digital application). Within two weeks of the baseline assessment, participants randomized to the intervention will receive the first of five telehealth consultations with an AEP (Fig. 1) over 3 months to develop and go through an exercise program individualised to their needs, physical activity levels, barriers, preferences and goals. The program will be designed with the purpose of participants utilising themselves, and/or with their family, peers, community sport and at school. Participants will be mailed simple exercise equipment to use at home for their program based on their preferences and needs such as resistance bands, skipping ropes and provided with a $50 (AUD) voucher to use on sport or physical activity equipment. The MERRIER exercise intervention is guided by exercise guidelines in childhood cancer [11], previous research (e.g. PICASSO Study [20] and the CanMOVE study which also adopts a flexible exercise prescription approach during the treatment phase for childhood cancer [42]), as well as the RePlay theoretical model for structured active play [43], and was co-designed with oncology health professionals, and childhood cancer survivors and their parents. The number of supervised consultations in the study (five) was selected to be in line with the number of government subsidised (Medicare) sessions with an AEP or physiotherapist available each year to Australians with a chronic condition.

During each consultation with the AEP, participants will discuss any challenges with the program, and adjustments will be made. The second component of each consultation is completing each prescribed exercise together, allowing for technique adjustments and questions. Where required, participants will be offered strategies on increasing their physical activity participation in line with the paediatric oncology exercise guidelines [11]. Physical activity programs will be structured and individualised based on the framework in Table 2.

**Table 2:** Framework for structure of exercise physiology consultations and factors for exercise prescription.

Exercise Guidelines	**Duration:** Education on paediatric oncology physical activity guidelines (“Move when you can”) [11]
	**Intensity:**Education on how to interpret exercise intensity using the modified borg rate of perceived exertion 1–10 intensity scale [33]. Education on light (1–3/10), moderate (4–5/10) and vigorous exercise intensities (≥6/10), and how to achieve these.
Type of exercise	**Aerobic, resistance, stretching, balance)**: Each program will include a component of age-appropriate aerobic, resistance, and stretching exercises, with balance exercises prescribed as needed. Exercises will be age-specific (e.g. games for young children, gym/structured programs for adolescence, sport teams for all. The exercises can be recommended do be done as an individual, or with siblings, parents, or friends. The exercise program will be provided using the physitrack software. Participants may also be recommended to use technology to promote adherence such as pedometers, digital exercise applications, or age-appropriate online videos.
Preferences based on enjoyment and goals	Exercises will be prioritised based on preferences and enjoyment (i.e. sport, play-based, dancing, games-based, cycling, walking, swimming, team sports) Discuss short and long-term goals (e.g. number of daily steps or minutes, assessment performance goals, return to sport or activity, reducing symptoms). If goal is not achieved, discuss why not and a formulate contingency plan.
Education	Each appointment will include an educational component with videos and discussion, which include: 1. Principles of exercise and cancer 2. Graded exercise: How to progress and regress 3. Activity pacing for fatigue 4. *Topic chosen by participant from*: managing peripheral neuropathy, social support, long-term maintenance, self-monitoring physical activity 5. Digital technology and local programs
Referral	At the conclusion of the intervention, the study team will recommend a local exercise professional (e.g. accredited exercise physiologist, physiotherapist, or community sport program) who can also assist with delivering a face-to-face exercise program locally, if they choose to. Participants will be encouraged to share their prescribed program with local health professionals and their school teachers, so their teachers are informed on any exercise considerations and any contraindications, who will be permitted to communicate with the research team for information regarding the program.

An example of the exercise program parameters is presented in Table 3, and further detail for each consultation is presented in Supplementary File 1. Each online consultation in the intervention group will be 45–60 ​min in duration. This is broken down into a 20-min discussion including: goal setting, discussing the prescribed physical activity program, and providing education. Following will be a 30-min supervised exercise session, which includes: (1) 5 ​min warm-up; (2) 20 ​min of exercise training consisting of strength/resistance, balance, and aerobic activities, and (3) 5 ​min cool-down consisting of full-body stretching. The AEP demonstrates each exercise, tailoring to address participants’ needs including exercise progressions (e.g. push-ups from wall to floor) or regressions (e.g. push-ups from floor to wall).

**Table 3:** Example of exercise program parameters, which may vary based on baseline level of physical function, fitness and exercise history.

Video Consult #	Consultation 1	Consultation 2	Consultation 3	Consultation 4	Consultation 5
Week	1	2 to 3	4 to 6	7 to 9	10 to 12
Theme	Adaptation	Individualisation	Progressive overload		Transition
Contents	Warm up, teaching about movements and equipment	Tailoring exercises to needs of individual, motor skills, strength, fitness	Strength, fitness, motor skills		Strength, fitness, motor skills
Resistance exercises	Body weight only (E.g. bridges)	Body weight only (e.g. knee push ups)	Introduce light resistance bands, hand weights or jumping	Combination of resistance exercise types	
Aerobic exercises	E.g. Dancing, bike riding, ball games	Introduction to circuit training	Introduction of vigorous intensity bouts		
Other exercises (e.g. introduce balance exercises as needed if balance/stability is an issue)	Static	Static	Dynamic	Eyes open/closed	Challenging
Sessions per week	2	2	3	3	3
Total number of exercises	3	3 to 4	4 to 5	5	5 to 6
Minutes per session	15	20	25 to 30	35	40
Rest time (sec)	90	90	75	60	60
Rating of perceived exertion (/10)	4	5	6	6 to 7	6 to 8

### 2.9 Education

Within each of the five AEP intervention consultations, there will be a discussion incorporating an educational topic about the role of exercise in childhood cancer. This will be delivered using video and written information resources and discussions on the topic which will be addressed to the parent, with age-appropriate language tailoring to the participant. This will facilitate further learning on behaviour change techniques and key exercise principles in cancer care. The education topics include (1) Principles of Exercise and Cancer, (2) Graded exercise: how to progress and regress, (3) Activity pacing for fatigue, (4) Digital technology and local programs, and (5) a topic chosen by participant from: managing peripheral neuropathy, social support, long-term maintenance, and self-monitoring physical activity. These education topics were developed by engaging with consumer families, and designed to build self-efficacy to equip participants with the behaviour change techniques to apply in their daily life, in line with the study primary aim. Specific skills learnt include self-monitoring, barrier management, planning, goal setting, growing social support, and building confidence and belief in their own ability to be physically active.

### 2.10 Control group

The control group will not receive an individualised exercise program during the control period, but instead will be emailed educational resources about the importance of physical activity after allocation into the control group including online resources developed by Exercise and Sport Science Australia [44] and the International Pediatric Oncology Guidelines [45]Control group participants will be offered a delayed-start opportunity to participate in the intervention after the 24 week follow-up (T2) assessment. This approach allows for ethical treatment and addresses any potential concerns about withholding treatment.

### 2.11 Assessing implementation: the reach, effectiveness, adoption, implementation, and maintenance (RE-AIM) framework

The reach, effectiveness, adoption, implementation, and maintenance (RE-AIM) framework will be used to evaluate the implementation of the MERRIER study into a community organisations usual operations from individual (i.e., survivors and families) and organisational levels [46]. Reach will be measured by the calculating the proportion and characteristics of individuals who were eligible and able participate, as well the proportion excluded and reasons for exclusion. Effectiveness measures of the co-primary outcomes (self-efficacy and objectively measured moderate-vigorous physical activity), and secondary outcomes (e.g., patient-reported outcomes and physical assessments) for group changes will be conducted between baseline (T0) and post-intervention (T1). Adoption will be measured by calculating the number of enrolled and completed participants by the total eligible, while qualitative interviews with research staff will be conducted iteratively to understand reasons for non-recruitment and drop-out mid-way through recruitment. Implementation will be measured by calculating the adherence (percentage of prescribed exercises completed) and compliance (percentage of exercise intervention consultations completed, and number of participants who complete >70 ​% of their prescribed exercises using the Physitrack exercise software), and listing adaptations made to the intervention during the study. Maintenance will be a measure of the co-primary and secondary outcomes at post-intervention (T1) and 24-week follow-up (T2) (i.e. >60 ​% of participants report a self-efficacy score and physical activity minutes (using self-report) equal to or higher than their baseline score at the 24-week follow-up (T2)). Fidelity checks will be carried out to ensure consistency and safety in the delivery of the online exercise intervention. Consultations will be recorded using Microsoft Teams and stored on a secure password-protected cloud-based network at The University of Sydney for quality assurance. Ten percent of exercise consultations will be randomly reviewed to observe and improve program delivery.

### 2.12 Semi-structured interviews

Semi-structured interviews in a sub-set of participants (n ​= ​10 parent and participant dyads) and research and community organisation staff (n ​= ​5) will be conducted using the Consolidated Framework for Implementation Research (CFIR) to identify barriers and enablers to program implementation (e.g. reasons for (or lack of) program maintenance, adherence) [47]. Interview outcomes will aim to understand adoption of the intervention at the setting level, understand staff participation, adaptations required, and whether the program (or specific components) were adopted in the long-term (at the follow-up (T2) timepoint, i.e. 3 months after completion of the program). Purposive sampling will be conducted to ensure diversity of age, gender, ethnicity, activity level, as well as cancer diagnosis for participants. Interviews will be conducted either online using Microsoft Teams or via telephone with trained study staff.

### 2.13 Sample size

In this study, a sample size of 52 patients is required to achieve 90 ​% power to detect a significant difference (p ​< ​0.05) of 10 ​min/day difference in moderate-vigorous physical activity measured using the Axivity AX3 accelerometer between the experimental and control groups at post-intervention (T1). This calculation was conducted using the Sealed Envelope™ online Power Sample Size Calculator tool [48], assuming a standard deviation of 11 ​min/day and a 15 ​% drop-out rate. Therefore, this study will enrol 60 childhood cancer survivors.

### 2.14 Statistical analysis

Descriptive statistics will be used to summarise participant demographic factors of age, sex, rural/urban, primary diagnosis of cancer, comorbidities, treatment received, as well as RE-AIM domains. We will perform generalised linear mixed models to evaluate effectiveness changes in outcome measures over time. To deal with the variance in effectiveness of implementation, we will employ multilevel modelling to examine differences in relation to reported physical activity levels and adherence to the exercise intervention by age. Quantitative data will be analysed using IBM SPSS software (Version 26.0, IBM Corp., Armonk, NY). Qualitative data will be transcribed using Microsoft Teams, imported into NVivo Version 12 for coding, and thematically analysed following Braun and Clarke's six-step framework [49]. Two independent authors will code the data to ensure reliability, with discrepancies resolved through discussion or consultation with a third researcher, if necessary. The analysis will be conducted iteratively, allowing for themes to emerge inductively from the data while considering deductive insights from the study objectives [50].

### 2.15 Patient and public involvement

The MERRIER study conception, delivery, assessments and implementation was developed by researchers, in collaboration with childhood cancer survivors (n ​= ​3) and parents of a survivor (n ​= ​10), as well as medical oncologists (n ​= ​4), nurses (n ​= ​3) and exercise professionals with experience in childhood cancer (n ​= ​4). Consumers were invited to provide input into the development of the program, study documentation and dissemination. Our team also engages with international leaders in the paediatric exercise oncology field throughout the entire project in terms of study design, exercise intervention details, and selection of assessments to continually improve the scientific rigour and exercise programme experience for participants.

### 2.16 Ethics and dissemination

Ethics approval was received from The University of Sydney Health Research Ethics Committee (2024/HE000391). Our research team will disseminate information regarding the MERRIER study via quarterly newsletters sent to our collaborators, participants and community organisations. Quarterly newsletters will include study updates on overall recruitment. Our research team will submit abstracts to present the findings at scientific conferences and publish as journal articles.

## 3 Discussion

This study protocol was developed to investigate the effectiveness and implementation of an online delivered exercise program on childhood cancer survivors’ physical activity levels and self-efficacy. The MERRIER study was developed in consultation with childhood cancer survivors, consumer parents, and paediatric oncology healthcare professionals with a goal of safely delivering exercise and discussing the associated benefits to children and their caregivers. The five supervised online consultations with the AEP in the trial reflects a real-world clinical offering in Australia under the Chronic Disease Management Plan from Medicare, thus has the potential for realistic clinical implementation. The findings will be compared to other online trials in progress including the iBoneFIT study [51] and IMPACT study (NCT04956133) which is conducted during the treatment phase with 16–36 online sessions versus 5, as well as other studies including those that used online gamified platforms [52]. Childhood cancer survivors may benefit from receiving remotely delivered exercise programs [7], due to the barriers they experience, reduced activity and fitness levels compared to their peers and the risk of medical late-effects. Given that exercise is not widely implemented into childhood cancer models of care, this highlights the need for more data on need, effectiveness, and implementation experiences in child and teenage groups.

Childhood cancer survivors arguably may benefit more than the general population from participating in personalised exercise programs due to their high burden of medical late effects experienced and large number of years to live after receiving cancer treatment [53,54]. The MERRIER study aims to address this inequity by implementing and evaluating remotely delivered exercise programs to children who have completed their cancer treatment. As one of few studies investigating remotely delivered exercise for childhood cancer survivors, findings will inform whether this method of delivery is beneficial at enhancing the physical activity levels, self-efficacy, fitness and HRQoL for children living beyond cancer.

The findings will be shared with professional and community networks across the paediatric oncology and exercise science fields. Building partnerships with community organisations will support the potential scale-up of health interventions including exercise. Collaborating with a community organisation using a hybrid implementation study design is a key strength of this study to examine factors and accelerate the traditional research-to-practice implementation timeline outside the conventional clinical setting (approximately 17 years [55]). Many childhood cancer community organisations have established partnerships with hospitals, providing avenues for families to receive community and social support. Collaboration and buy-in from community organisations may be integral to implement health interventions including exercise support in becoming a component of standard supportive cancer care given lack of capacity for hospital-based services to support all patients in-need. For hospitals that provide limited or no exercise programs, they could provide information in outpatient clinics about relevant community programs (such as the MERRIER study), while having treating medical teams endorsing physical activity could provide parents with higher confidence about the safety and benefits for their child, which can then promote greater physical activity uptake.

The MERRIER study aims to generate a greater understanding of determinants of implementation of exercise in a community setting, which will assist bridging the gap from current clinical practice which generally lacks exercise support, to implementing exercise support across communities, schools, and clinics. Bridging these settings is critical to implement the role of the exercise professional within cancer care. Connecting exercise professionals with oncology expertise to patients ensures that evidence-based exercise programs are appropriately delivered to meet each patients’ needs. Ultimately, the MERRIER study aims to deliver exercise to patients and families in conjunction with a community organisation with the goal to equip children living beyond cancer and their families with the knowledge, confidence and behaviour strategies to optimise their exercise to manage their fitness, health, and HRQoL.

## Consent to participate

Informed consent was not required for this protocol paper.

## Availability of data and material

There are no datasets generated from this protocol.

## Code availability

There are no datasets generated from this protocol.

## Authors’ contributions

DM conceived and received funding to support the study. All authors contributed to the study design. DM drafted the protocol with input from all authors. DM led the manuscript with input from all authors.

## Ethics approval

The study was approved by The University of Sydney Health Research Ethics Committee (2024/HE000391).

## Consent for publication

Not applicable.

## Funding

David Mizrahi and Lauren Ha are supported by fellowships from The Kids Cancer Project. Alexandra Martiniuk is supported by an Australian National Health and Medical Research Council (NHMRC) Investigator Grant (1195086). Ben Smith is supported by a Cancer Institute NSW Career Development Fellowship (2021/CDF1138).

## Declaration of competing interest

The authors have no competing interests to declare that are relevant to the content of this article.
