# Supplementary material for: Implementing an online-delivered exercise program for childhood cancer survivors: A hybrid effectiveness-implementation protocol for the MERRIER study
Source: JSAMS Plus. 2025 Mar 12;5:100095. doi: 10.1016/j.jsampl.2025.100095 (PMC13008456; doi:10.1016/j.jsampl.2025.100095)
Supplement: Multimedia component 2 [file mmc2.docx]

**Table 4.** Examples of exercise adaptations based on common adverse side-effects, transcribed from the Pediatric Oncology Exercise Manual [1]

| **Side-effect** | **Exercise program adaptation example** |
| --- | --- |
| Chemotherapy-induced peripheral neuropathy (CIPN) | CIPN symptoms including tingling and numbness in the hands and feet may impact walking, running and balance. Participants with CIPN may require additional balance training that is both static and dynamic, functional exercises, and lower leg strengthening. |
| Cardiotoxicity | Participants who develop cardiotoxicity may require additional clearance from their clinical team or general practitioner to continue in the study. They may require reductions in intensity, in particular reducing higher intensity resistance training, and encouraging avoiding breath holding (Valsalva manoeuvre). |
| Osteonecrosis | Ensure prescribed exercises are pain free and within the impacted joints range of motion. Encouraged a combination of progressive resistance training, aerobic and flexibility exercises. Caution movements that increase pain levels, high impact exercises and adhere to any orthopaedic weight-bearing precautions |
| Infections | Avoid exercise if fever >38 degree Celsius. Practice caution if participant is experiencing severe neutropenia. If able to exercise, hygiene control should be practiced, such as cleaning hands and cleaning exercise equipment. Water-based exercise and high-intensity exercises may be avoided. Aerobic exercise should be at low to moderate intensity, while resistance exercise can still be encouraged. |

1. Chamorro-Viña, C., M. Keats, and S. Culos-Reed, *Pediatric Oncology Exercise Manual - Professional Version.* 2014: Health and Wellness Lab, Faculty of Kinesiology, University of Calgary.
